# IgG4-Related Hypophysitis: Case Report and Literature Review

**DOI:** 10.7759/cureus.907

**Published:** 2016-12-01

**Authors:** Lauren Decker, Angela M Crawford, Gamaliel Lorenzo, Martina Stippler, Konstantin N Konstantinov, Karen SantaCruz

**Affiliations:** 1 Pathology, University of New Mexico School of Medicine, Albuquerque, New Mexico; 2 Department of Surgery, University of Maryland; 3 Department of Radiology, University of New Mexico School of Medicine, Albuquerque, New Mexico; 4 Neurosurgery, Beth Israel Deaconess Medical Center; 5 University of New Mexico School of Medicine, Albuquerque, New Mexico

**Keywords:** plasmacytic hypophysitis, lymphocytic hypophysitis, igg4 related disease

## Abstract

IgG4-related hypophysitis is a rare, inflammatory process of the pituitary that mimics more commonly seen pituitary tumors. We report a case of IgG4-related hypophysitis in a 16-year-old female with diabetes insipidus who was found to have IgG4-related hypophysitis based on tissue diagnosis. This entity has not been previously described in a pediatric patient. Recognition of certain inflammatory processes of the pituitary may lead to alternative means of diagnosis and medical management without a biopsy.

## Introduction and background

IgG4-related disease (IgG4-RD) is a rare, newly recognized, multi-organ disease characterized by a tendency to form tumefactive lesions in multiple sites throughout the body. Pituitary involvement, IgG4 hypophysitis, is less commonly reported and, when described, is usually associated with IgG4-RD elsewhere in the body. In this case report, we present a pediatric patient who presented with diabetes insipidus and an isolated suprasellar mass which was subsequently resected revealing a pathologic diagnosis of IgG4 hypophysitis. This is the first report of IgG4 hypophysitis in a pediatric patient. Given that the treatment of IgG4 hypophysitis is drastically different than treatment for other more common pituitary tumors within this population, it is important to consider this entity in the differential in order to prevent unnecessary invasive procedures and subsequent morbidity.

### Case report

**Figure 1:**
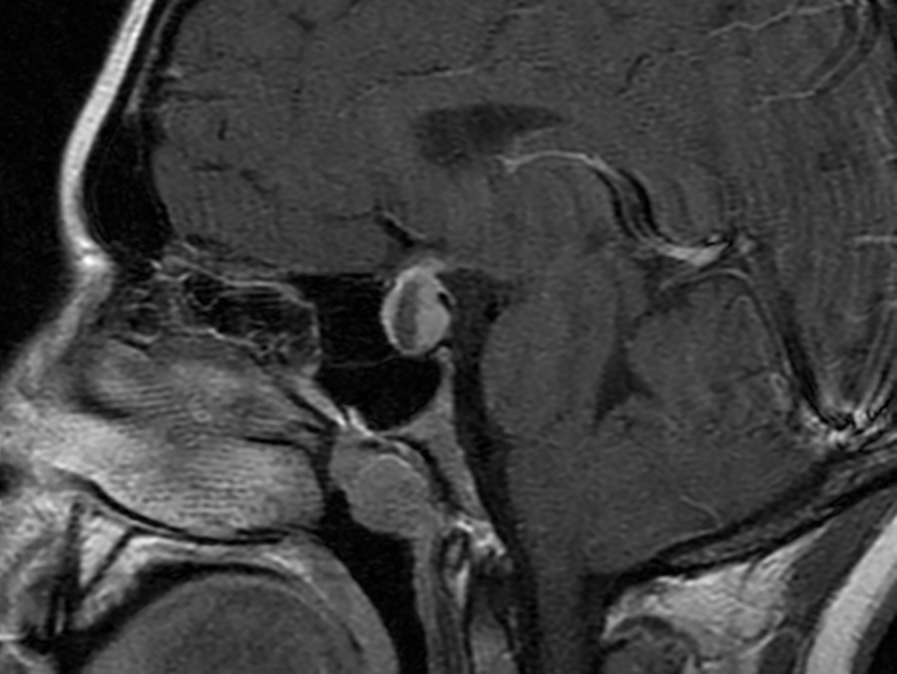
Neuroimaging of Pituitary Mass T1 MRI with contrast showing a 2 cm ring-enhancing suprasellar mass with effacement of the optic chiasm.

A 16-year-old female presented to our endocrinology, otolaryngology, and neurosurgery clinics for an evaluation of a suprasellar lesion. She had presented three months previous to an outside endocrinologist for excessive polyuria and polydipsia. She was found to have a serum osmolality of 292 mOsm/kg and a urine osmolality of 50 mOsm/kg, and was given a diagnosis of diabetes insipidus. During the workup, magnetic resonance imaging revealed a 2 cm peripherally enhancing, partially cystic sellar/suprasellar mass causing compression of the optic chiasm (Figure 1).

She was referred to our institution for definitive treatment of what was thought to be most likely a craniopharyngioma. The patient agreed to participate and was explained the nature and objectives of this study, and informed consent was obtained. No reference to the patient's identity was made at any stage during data analysis or in the report.

When she presented at our institution, she had previously been placed on DDAVP 0.05 mg twice a day, which had largely resolved her previous symptoms although she did complain of occasional headaches. There were no visual changes, sicca symptoms, rashes, vocal changes, smell or taste changes, amenorrhea, galactorrhea, Raynaud’s, or photosensitivity. Her past medical history was significant for atopic dermatitis controlled with steroid creams and right hearing loss since age five. On examination, her visual fields and acuity were intact, and neurological examination was unremarkable. There was no corneal dryness or ulceration, and the oral mucosa showed adequate saliva pool and no sores. Parotid glands were not tender or enlarged, and the thyroid gland was not palpable.

Laboratory testing showed free T3, T4, thyroid-stimulating hormone, and a random serum cortisol were all within normal limits. Serum prolactin and growth hormone levels were normal. Follicle-stimulating hormone and luteinizing hormone were appropriate. Urine specific gravity was normal as well.

Computed tomography performed at our institution showed a low attenuating sellar/suprasellar lesion similar to what was seen with MRI imaging at the outside institution. No calcifications were appreciated. No cavernous sinus involvement was found. Scattered white matter abnormalities were present though stable and thought to be unrelated to the pituitary findings.

Based on imaging and presentation, the differential included a craniopharyngioma, hypophysitis, and less likely a pituitary adenoma. Clinical signs and symptoms suggestive of granulomatosis with polyangiitis (GPA) (Wegener's) were not present. Because of the possibility of mass effect on the optic chiasm and radiologic findings consistent with craniopharyngioma, namely the solid/cystic appearance and the peripheral enhancement of the lesion, an endoscopic transsphenoidal resection of the pituitary was performed. The lesion was found to be encapsulated and consisted of a white-tan diffluent material. The lesion was completely resected from the sella and sent, in aggregate, for pathologic evaluation.

**Figure 2:**
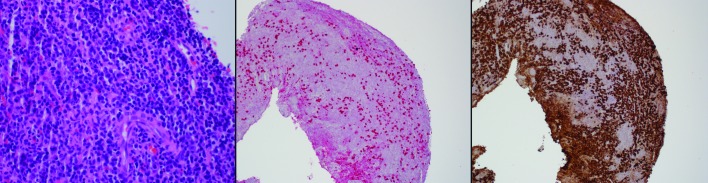
Pituitary Histopathology Panel A: Hematoxylin- and eosin-stained section at 60x oil showing abundant plasma cells intermixed with occasional lymphocytes. Panel B: IgG4 immunostaining (red chromogen) at 10x revealing that a large percentage of the plasma cells are IgG4 positive. Panel C: IgG4 immunostaining at 10x confirming that nearly all of the inflammatory cells are plasma cells.

Sections of the lesion showed extensive infiltration of mature plasma cells intermixed with lymphocytes (Figure 2). Sections were immunostained for both IgG4 and IgG, and the calculated ratio approximated 25%. However, the 50 plasma cells/high-power field meets consensus criteria for diagnosis of the plasmacytic hypophysitis even in the absence of a ratio exceeding 40% IgG4/IgG or (storiform) fibrosis. The plasma cells numbered over 50 per high-powered field. By kappa and lambda in-situ hybridization, the plasma cells were polytypic, ruling out a clonal process. No significant fibrosis was noted. These findings were considered consistent with a diagnosis of IgG4 hypophysitis.

Postoperatively, the patient experienced a small CSF fluid leak which was repaired surgically without issue. She was discharged from the hospital on steroid replacement and DDAVP. Given the diagnosis, the patient was referred to Rheumatology for follow-up. She was found to have no obvious sign or symptoms of systemic involvement of IgG4-RD. An IgG4 level was performed and was normal; however, the patient was receiving chronic steroid replacement therapy at that time. Unfortunately, there have been no follow-up visits at our institution since that time.

## Review

IgG4-related disease (IgG4-RD) is a newly recognized, fibroinflammatory disease that has been shown to affect almost every organ of the body, with preference for the pancreas, salivary gland, orbital tissue, lymph node, lung, and kidney. It is characterized by tumorous swelling of affected organs, prominent lymphoplasmacytic infiltrate rich in IgG4-positive plasma cells, and high serum IgG4 concentrations. IgG4-RD can also cause diffuse infiltrative lesions presenting without constitutional symptoms, which usually makes the diagnosis incidental and based upon radiographic findings or examination of pathological specimens. Spontaneous improvement is very rare, and the majority of cases show slow and indolent progression. IgG4-RD affects mostly middle-aged and elderly men, but the male-female ratio is balanced in patients with predominantly head and neck involvement. It is important to differentiate IgG4-RD from malignancies with mass-forming lesions and some immune-mediated diseases (e.g.Churg-Strauss syndrome, multicentric Castleman’s disease, sarcoidosis, Sjogren’s syndrome) by additional histopathological examination. Granulomatosis with polyangiitis (GPA) (Wegener's) may mimic IgG4-related disease and is an important consideration in the differential diagnosis [1-3]. The pathogenesis of IgG4-RD is unknown but is likely related to autoimmunity or chronic infection [4]. At this time, elevated serum IgG4 or IgG4-IgG ratio is found in up to 70% of patients with IgG4-RD, but data regarding the use of their serial measurements as disease activity markers are uncertain [5]. So far, there is no evidence to suggest that the IgG4 autoantibodies are directly pathogenic in IgG4-RD. This is in sharp contrast with certain immune-mediated conditions (pemphigus vulgaris and foliaceus, thrombotic thrombocytopenic purpura, and some cases of childhood membranous glomerulonephritis) in which autoreactive IgG4 autoantibodies induce direct damage by binding their cognate antigens [6].

The recognition of IgG4-RD involvement of the pituitary created a new category within the broad term of hypophysitis, which is used to describe inflammatory disorders of the pituitary.  Within this spectrum, many types of hypophysitis have been identified including two more common forms (lymphocytic/autoimmune and granulomatous) and several more rare forms (xanthomatous, necrotizing, and plasmacytic/IgG4-related). Interestingly, while lymphocytic hypophysitis is also pathologically characterized by a lymphoplasmacytic infiltrate, there is a predominance of lymphocytes (B and T cells) rather than plasma cells [7]. Additionally, the demographics of the two diseases are very different--lymphocytic hypophysitis being described in young adult females (mean age at presentation 38 years; female to male ratio of 3:1) and IgG4-related occurring in older males [8]. It is not certain whether these entities are distinct or lie on the same histopathologic spectrum without specific assessment of IgG4 levels on serology or immunohistochemistry. Intracranial IgG4-RD presents as pachymeningitis or hypophysitis and generally does not affect the brain parenchyma. When the pituitary gland or stalk is affected, the most commonly reported outcomes include hypopituitarism, diabetes insipidus, or local mass effect [9].

A review of the 18 reported case reports of IgG4-related hypophysitis [8, 10-24] shows that all but one patient presented with a form of hypopituitarism, including central diabetes insipidus, hypogonadism, hypothyroidism, and hypocortisolism (Table 1). Many patients also had symptoms related to mass effect.

**Table 1:** Summary of Demographic, Radiographic and Clinical Information from a Review of 18 Previously Published Cases of IgG4-Related Hypophysitis.

Study	Age, Gender, Race	Clinical Symptoms	Laboratory	CNS Imaging (modality)	Biopsy Source	Other Organ Involvement	Treatment, Response
Van der Vliet et al., 2004 [10]	66, F, not reported	Nausea, headache, swollen salivary gland	Hypothyroidism, increased serum IgG4	Sellar mass (CT)	Submandibular mass	Present	Prednisone, marked
Yamamoto et al., 2006 [11]	70, M, Japanese	Swollen salivary glands, sexual dysfunction	Hypogonadism, increased IgG4	Enlarged pituitary stalk (MRI)	Submandibular mass	Present	Prednisolone; marked
Tanabe et al., 2006 [12]	71, M, Japanese	Fatigue, weight loss, polydipsia, swollen salivary glands	Panhypopituitarism, increased IgG4	Swelling of pituitary (MRI)	Salivary gland and retroperitoneal mass	Present	Hydrocortisone, marked
Ralli et al., 2007 [13]	67, M, not reported	Generalized weakness	Panhypopituitarism, increased IgG4	Enlarged stalk and pituitary (MRI)	Pancreas	Present	Prednisone, marked
Wong et al., 2007 [14]	77, M, Chinese	Blurred vision	Hypogonadism, increased IgG4	Pituitary mass (MRI)	Pituitary, pancreas, gallbladder	Present	Hydrocortisone, not known
Isaka et al., 2008 [15]	55, M, Japanese	Fatigue, polyuria/polydipsia, nasal discharge	Diabetes insipidus (DI)	Enlarged stalk (MRI); Mass in sphenoidal, maxillary, frontal sinuses (CT)	Paranasal mass	Present	Prednisone, marked
Tsuboi et al., 2008 [16]	62, M, Japanese	Fatigue, weight loss, fever	Hypocortisolism, hypothyroidism, DI	Enlarged stalk (MRI);	Lung	Present	Glucocorticoid, good
Osawa et al., 2009 [17]	74, F, Japanese	Fatigue, polydipsia	Panhypopituitarism	Swelling of pituitary (MRI)	Pituitary	None	Glucocorticoids; marked
Hori et al., 2010 [18]	70, M, Japanese	Fatigue, thirst, nausea	DI, adrenal insufficiency, increased IgG4	Enlarged pituitary and stalk (MRI)	Salivary gland, liver, lung	Present	Hydrocortisone, marked
Haraguchi et al., 2010, case 1 [19]	74, F, Japanese	Fatigue, anorexia, polyuria, polydipsia	Panhypopituitarism, DI, increased IgG4	Swelling of pituitary and stalk (MRI)	None	None	Prednisone, marked
Haraguchi et al., 2010, case 2 [19]	68, M, Japanese	Polyuria, polydipsia	DI	Enlargement of stalk (MRI)	Retroperitoneal	Present	Prednisolone, good
Leporati et al., 2011 [8]	75, M, Caucasian	Headache	Panhypopituitarism	Enlarged pituitary, sphenoid sinus mass (MRI)	Pituitary	Present	Prednisolone, good
Patel et al., 2011 [20]	55, M, Native American	Weight loss, fever, headache, fatigue, polyuria, diplopia	Panhypopituitarism, increased IgG4	Enlarged pituitary (MRI)	Kidney, retroperitoneal, lymph nodes, lacrimal gland	Present	Prednisone, marked
Hsing et al., 2013 [21]	66, M, Chinese	Weight loss, nausea, vomiting	Hypogonadism, hypoadrenalism	Sellar mass (MRI)	Posterior mediastinal mass, pituitary mass	Present	Prednisolone, good
Hattori et al., 2013 [22]	55, M, Japanese	Bitemporal hemianopsia	Increased IgG4	Enlarged pituitary gland (unknown)	Pituitary	None	Prednisolone, good
Caputo et al., 2014 [23]	40, M, Vietnamese	Lethargy, polyuria, polydipsia	Panhypopituitarism, DI	Pituitary lesion (MRI)	Lacrimal gland, pituitary	Present	Prednisolone, incomplete; azathioprine, good
Kanoke et al., 2013, case 1 [24]	53, F, not reported	Headache	Hyperprolactinemia	Pituitary mass (MRI)	Pituitary with dura	Present	Hydrocortsone, good
Kanoke et al., 2013, case 2 [24]	27, F, not reported	Hx of germinoma, headache and fatiguability	Panhypopituitarism and hypothyroidism	Sellar lesion (MRI)	Pituitary with dura	None	Hydrocortisone, unknown
Present case	16, F, unknown	Polyuria, polydipsia	DI	Sellar mass (MRI)	Pituitary	None	Hydrocortisone unknown

Demographic details of the 18 reported cases demonstrated a mean age of 62.5 years (range 27-77) and a male predominance 13/18 (72%). The majority of the cases have been described in patients of Asian descent; however, this may be reflective of the increased awareness of IgG4-RD within the community. Each of these cases had other organ manifestations either synchronously or metachronously except two cases having hypophysitis as the sole manifestation in a 74-year-old female [17] and 55-year-old male [22]. This allowed for some diagnoses to be made based on biopsies performed on areas that were more easily accessible and less invasive, such as the salivary gland, with concurrent imaging and serum IgG4 levels. Only a minority of the patients required a pituitary biopsy for definitive diagnosis. Once diagnosed, all patients were treated with steroids with or without hormone replacement therapy depending on their initial presentation. All patients showed clinical improvement after the start of steroid therapy, some with a complete resolution of symptoms. Several patients continued to need hormone replacement therapy after the resolution of their hypophysitis suggesting damage to the pituitary may be permanent in some cases.

Diagnosis of IgG4-related hypophysitis can be made a variety of ways. Pituitary biopsy is the most definitive; however, a biopsy can be invasive and unnecessary in certain cases. Diagnosis of IgG4-related hypophysitis can also be suggested based on biopsy-proven IgG4-related disease in other organs when classic imaging findings of hypophysitis are present, including homogeneously enhancing sellar mass/thickened pituitary stalk. A guideline for a histologic diagnosis of IgG4-related disease has been previously described in a consensus statement on the entity and includes a dense lymphoplasmacytic infiltrate, storiform fibrosis, obliterative phlebitis, and an increased number of plasma cells (Table 2).

**Table 2:** Consensus Diagnostic Criteria for IgG4-related Hypophysitis (Deshpande et al., 2012)

Diagnostic Criteria for IgG4-Related Hypophysitis	
1	Pituitary Histopathology
	Mononuclear infiltration of the pituitary gland, rich in lymphocytes and plasma cells, with more than 10 IgG-positive cells per high-power field
2	Pituitary MRI
	Sellar mass/thickened pituitary stalk
3	Biopsy-Proven Involvement in Other Organs
	Association with IgG4-positive lesions in other organs
4	Serology
	Increased serum IgG4 (>140 mg/dl)
5	Response to Glucocorticoids
	Shrinkage of the pituitary mass and symptom improvement with steroids
Diagnosis of IgG4-related hypophysitis is established when any of the following is fulfilled:	
	1 2 and 3 2, 4, and 5

For some organs, obliterative phlebitis and storiform fibrosis are uncommon, and consensus histologic criteria for these features with pituitary involvement have not been described [25]. The number of plasma cells per high-power field suggested as an appropriate cutoff varies per author but ranges from 10 to 50, with 50 plasma cells per high-power field being highly specific. A secondary diagnostic tool is the IgG4+ to total IgG+ plasma cell ratio, with over 40% as a proposed cutoff value [25]. If no biopsies are available, a third option for diagnosis includes classic imaging, increased serum IgG4 levels (>140 mg/dl), and a good response to glucocorticoids as seen by symptom improvement and shrinkage of the pituitary lesion.

While there is no standardized regimen for steroid therapy for this disease, a dose of 0.6 mg/kg/day of prednisolone is most often used. This should be continued for 1-2 months, with a taper of 5 mg per week. If relapse should occur, the physician may consider a maintenance dose for an extended period of time, up to three years, or a combination of immunosuppressants [26-27].

The present case differs from the reported cases of IgG4-related hypophysitis in several important aspects. The first aspect which makes this case interesting is the lack of convincing systemic features. Most patients with this disease have concurrent or previous involvement of other organs. Her lack of systemic involvement made it difficult to diagnose hypophysitis without tissue examination. Imaging studies were also misleading in this patient, with features suggestive of a neoplastic process, particularly solid and cystic components that would be most commonly seen in craniopharyngioma.

Another unusual aspect is the age of the patient. There have been no reported cases of IgG4-related hypophysitis in a patient less than 27 years old. While rare, IgG4-RD has been described in the pediatric population, usually within the context of autoimmune pancreatitis. Often these patients have additional systemic manifestations including retroperitoneal fibrosis, sialadenitis, and mediastinal adenopathy [28]. It is likely that other cases of IgG4-related hypophysitis have occurred in this age group and have been reported as different entities, or that treatment of co-existing systemic symptoms effectively treated the hypophysitis. The most recent cases described by Kanoke et al., illustrate this point--two adult patients were diagnosed as autoimmune hypophysitis (case 1) and granulomatous hypophysitis (case 2), but met histologic criteria for IgG4-related hypophysitis with increased IgG4+ and IgG ratios of 50% and 40%, respectively [24]. Recent literature has suggested that not infrequently there are cases of hypophysitis with IgG4 plasma cells that are not part of the systemic syndrome [29].

## Conclusions

IgG4-related hypophysitis is a rare condition not previously reported in a pediatric age group. The clinical presentation is similar to that for other inflammatory conditions of the pituitary. The decision to biopsy a pituitary mass in the teenage population is often straight forward for cases of a tumor; however, inflammatory processes which create a mass effect can sometimes be treated without a biopsy. This relies on the physician’s ability to recognize the possibility of rare, inflammatory processes such as IgG4 hypophysitis and having a knowledge of alternative ways to confirm the diagnosis. Avoiding a biopsy can decrease morbidities associated with the procedure and may avoid the need for long-term hormone replacement. Because this disease process has not yet been described in the pediatric population, and due to lack of systemic organ involvement or serum IgG4 levels to indicate a possible IgG4-related disease process, a biopsy was necessary to rule out a more common pituitary tumor and to achieve the diagnosis of IgG4-related hypophysitis. Recent literature has suggested that not infrequently there are cases of hypophysitis with IgG4 plasma cells that are not part of the systemic syndrome. Although it appears likely that this patient is not at risk for further involvement of multiple sites, a definitive biopsy has allowed the patient to seek close clinical follow-up by a rheumatologist.
